# 

**Published:** 1883-09

**Authors:** 


### Pamphlets.

---

A Contribution to the Study of Neglected Lacerations of the Cervix Uteri and Perineum. By Thomas A. Ashby, M.D., Professor of Obstetrics Woman's Medical College, Baltimore, etc., etc. Read before the Clinical Society of Maryland, May 4th, 1883.

Observations on the Management of Enteric Fever According to a Plan Based Upon the So-called Specific Treatment. Read before the College of Physicians of Philadelphia, January 3d, 1883. By James C. Wilson, Physician to the Jefferson Medical College Hospital. Extract from the Transactions, Third Series, Vol. VI.

A Report on Laceration of the Cervix Uteri. By T. B. Harvey, M.D., Prof. in Medical College of Indiana, etc.

---

THINGS WORTH KNOWING.—That wild mint will keep rats and mice out of the house.

That flowers and shrubs should be excluded from a sick chamber.

That lime sprinkled in fire-places during the summer months is healthy.

That a little water in butter will prevent it from burning when frying.

That oil paintings hung over the mantlepiece are liable to wrinkle with the heat.

That pennyroyal distributed in places frequented by roaches will drive them away.

That leaves of parsley, eaten with a little vinegar, will prevent the disagreeable consequences of a tainted breath by onions.

### INDEX TO VOLUME II.

Atlanta Medical College.......... 501
The Atlanta Medical Society.... 30
85, 158, 229, 334, 434, 469
Alcoholic Anæsthesia ............ 109
A Plea for the Education of Feeble-minded Children ....... 113
Abortive Treatment of Mammary Abcesses and the Cure of Fissured Nipples............ 14
A Case of Hemorrhagic Malarial Fever.. ........................ 77
A Word on the Treatment of Syphilis.............................. 615
Artificial Illustration of Percussion Sounds........................ 364
Artificial Human Milk............ 385
A Suggestive Contrast............ 565
Action of Alkalies on Pepsin... 48
A Word to the Wise............... 698
American Gynecology from an English Standpoint.............. 32
Atlanta's Potable Waters....627, 687
756
A New Danger to the Race...... 562
A Palatable Cough Mixture...... 555
A Valuable Sign of Death........ 548
As Others See Us, American Nervousness.. ...................... 549
Adulterated and Unreliable Drugs............................... 498
An Ethical Incident............... 679
A Method of Rendering the Skin Insensible in Operations without Chloroform........ ........... 670
A Furious Anti-Vivisectionist... 669
Adulteration of Bismuth Salts.. 489
Analysis of Urine.................. 635
A Case of Cerebro-Spinal Meningitis or Cerebro-Spinal Fever.................................. 329
An Unusual Case of Hæmaturia ................................... 332
A Winter Cold...................... 241
American Med. Association...... 610
Assafœtida in the Treatment of Abortion..... ...... .............. 620
Best Methods of Treating Operative Wounds........................ 341
Blood Poisoning.................... 286
Better than Vaccination, if True 570
Bismuth a Specific for Concrum Oris.................................. 676
Bacteria....... ......... ................ 165
Bright's Disease................335, 670
Bromide of Ethyl................... 290
Books......56 118, 183, 247, 311, 379
444, 504, 575, 637, 702, 762
Catarrhal Conjunctivitis—Sore Eyes........ ........................ 513
Chloroform Water......... ......... 366
Correspondence.....53, 374, 442, 574
683
Chemistry.............................. 566
Carbolic Acid in the Treatment of Wounds......................... 301
Castor Oil and Glycerine......... 750
Clinical Lecture on the Causes of Sterility and their Treatment .......... .................... ...... 648
Coffee and Its Effects............... 671
Carbolic Acid Poisoning by Injections............................... 672
Case of Ovariotomy in which the Expanded Bladder was Wounded............. .......... 619
Cauterization of the Clitoris in Hysteria............................. 620
Cincinnati Board of Health...... 701
Cotton Seed Oil in Pharmacy... 102
Contagiousness of Pulmonary Consumption...................... 111
Cure of Glycosuria and Diabetes Mellitus by Bromide of Potassium ............................. 111
Comparative Action of the Bromides.................................. 177
Death from the Sting of a Bee.. 747
Detection of Stone in the Bladder...................................... 748
Doctors.................................. 749
Dyspeptic Vertigo................... 177
Darwin............. .................... 48
Dr. J Thad. Johnson............... 702
Decomposing Organic Matter—Annual Address................. 539
Diphtheria............. ............ 700
Diffuse Non-Vascular Cornitis,.. 465
Darwin's Charity.................... 368
Ergot as a Preventive of Aural Troubles.............................. 747

Excision of Chancre as a Means of Aborting Syphilis............ 239
Eupatorium Perfoliatum.......... 554
Exercises on Chapter First of Chemistry............................ 625
Errata.................................... 636
Exercises on Chapter Second of Chemistry............................ 696
Experimental and Microscopical Researches in Traumatic Injuries of the Liver and Hepatic Abscess........................ 1
Examination of the Naso Pharynx in Children.................... 44
Fashion in Medicine................ 168
Festina Lente.......................... 115
Foreign Bodies in the Ear........ 35
Good Remedies Out of Fashion 737
Gynecological Points .............. 618
Heaton's Operation.................. 42
Hot Baths in Puerperal Convulsions.................................. 92
Hypertrophied Tonsils........... 101
Hypodermic Medication........... 449
How to Administer Santonine .. 553
Items.......63, 125, 189, 252, 319, 383, 447, 510
Instruction to German Midwives.................................. 241
Idiocy and Its Treatmeut......... 236
Intra-Uterine Injections in the Treatment of Puerperal Septicæmia............................ 484
Infantile Paralysis................ 338
Induced Abortion.................... 573
Iodine in Erysipelas................ 243
Intra-venous Injection of Common Salt.............................. 671
Iodoform in Diphtheria............ 681
Infectiousness of Tubercle....... 87
Local Anæsthesia.................... 551
Lead Poisoning from a New Cause.................................. 554
Ladies' Doctors...................... 299
Leprosy in the United States.... 641
Louisville Medical News.......... 702
Mistake by a Druggist; Damages 367
Medical College of S. Carolina. 470
Medical Association of Georgia 499
Malaria and Scrofula as Respective Causes of Acute and Chronic Skin Diseases.......... 181
Medical Diploma Scandal........ 107
Medical Empiricisms—Homœopathy and Codes.................. 654
Modern Therapeutics............... 105
Membranous Dysmenorrhæa..... 112
Medical Fashions .................... 179
Medical Society of London...... 474
McDaniel's Method of Artificial Respiration.............................. 614
Medical Morality...................... 47
Masturbation as an Etiological Factor in the Production of Gynic Diseases..................... 680
Menopause.............................. 620
Meningitis in Children............. 619
Manner of Putting up Journals for the Mails........................ 634
Moral Insanity and Imbecility.. 243
Measuration of the Chest......... 242
Non-Professional Criticism in Medical Matters.................. 117
Nymphomania and Hemorrhoids 308
Note on the Sulpho Carbolates.. 493
On the Detection of Bacilli of Tubercle in the Breath of Consumptive Patients............... 488
Obituary.................................. 500
One of the Abuses of Carbolic Acid...................................... 525
One of the Causes of Hæmoptysis.................................... 602
Our Annual Volume of Transactions................................ 372
Obstetrical Forceps.................. 46
Obstetrical Expedient ............. 742
Objectionable Anæsthesia........ 48
Overwork................................ 725
Pamphlets ......61, 124, 188, 251, 318, 383, 446, 508, 576, 640, 703, 764
Painless Synovial Effusions...... 234
Pay Up.................................... 761
Practical Chemistry for Students and Physicians.................... 571
Practical Chemistry for Students and Physicians, continued from June No................ 621
Practical Chemistry for Students and Physicians—Chapter Second. Continued from July No................................ 689
Practical Chemistry for Students and Physicians............ 751
Poisoning from Eating Plum Stones.................................. 749
Pop-Corn in the Vomiting of Pregnancy............................ 360
Publisher's Announcement...... 442
Protection from Venereal Disease...................................... 552
Pelvic Cellulitis........................ 65
Politics and Medicine............... 47
Pseudo-Specialism................... 50
Prospective.............................. 49
Pomade in Comedones............ 112

Precautionary Measures in the Use of Pressure by the Tampon, etc. 705
Present Status of Antiseptic Surgery 161
Quinine a Cause of Insanity 554
Quackery in America 352
Recent Changes in Medical Instruction 735
Reflex Irritation 744
Report of the Section on Practice for the Second Congressional District 556
Removal of the Secundines after Abortion 552
Rapid Dilitation of the Cervix Uteri 618
Rational Treatment of Menorrhagia 93
Rates of Mortality in Foreign Cities 672
Report of a Case of Battey's Operation 592
Removal of a Vesical Calculus 354
Renal Calculus 434
Specialties 110
Sewer Traps and Disease Germs in Sewer Air 37
Salts of Salicylic Acid in Acute Rheumatism 175
Some Facts about Feeble-minded Children 321
Simulated Stricture of the Œsophagus 334
Septicæmia 337
Styptics in General Surgery 344
Spray in Abdominal Surgery 365
Suppurating Synovitis of the Knee 346
Spasmodic Muscular Contractions, and Strychnine in the Treatment 435
Stricture of the Rectum 435
Salutatory 494
Suicide and Homicide Under Insidious Forms 578
Syphilis of the Spinal Cord 362
Sanitary Law 568
Sterility and Stenosis 617
Some New Discoveries in Regard to Erysipelas 606
Sassafras in Rhus Poisoning 621
Specific for Singultus 699
Sub-cutaneous Injections of Ether 39
The New Year 244
Therapeutic Effects of Hyoscymine 238
The Annual Report of the Board of the National Board of Health 246
The Outlook of Medicine and Surgery. No. 2 257, 577, 665
The Treatment of Hydrocele by Injections with the Hypodermic Syringe without Evacuation of the Sac 279
Taliaferro's Hard Rubber Intra-Uterine Stem Pessary 712
Tuberculosis 286
True Specialism 309
The Fourth Annual Report of the State Board of Health of Illinois 310
The Prevention and Cure of Cholera Infantum, So-called 439
Treatment of Rheumatism by Blisters 307
Tonsilotomy 357
Transactions of the Medical Association of Georgia 572
Typhoid Fever with Intestinal Hemorrhage and Relapse 336
Treatment of Partial Trichiasis by Electrolysis 348
Treatment of Icterus Neonatorum 351
The Dangers of Amateur Doctoring 363
The State Board of Health of Illinois 369
The Critics of the Committee on Revision of the New Pharmacopœia 371
The Quintessence of Absurdity 368
The State Medical Society of New York and the New Code 373
The Board of Health of the State of Georgia 437
The Approaching Meeting of the State Medical Association 441
Treatment of Syphilis 476
The Treatment of Diphtheria 487
Therapeutic Use of Clay 492
The Medical Code 495
To Detect Metalic Impurities in Natural Streams 545
The Pric Volta 554
To Detect Starch in Milk 555
The Contagiousness of Tuberculosis 286
Tracheotomy 308
Treatment of Neuralgia 302
The Frequent Repetition of Doses 599
That Instantaneous Light 575

The Combat Deepens — The Codical War in New York.... 575
Treatment of Placenta Previa... 621
The Picycle and Tricycle for Physicians.......................... 631
The Bromides........................ 633
The Maryland Med. Journal. ... 637
The Medical Bill............. ...... 636
The Treatment of Post-Partum Hemorrhage........................ 673
The Dangers of Illicit Drinking 683
Treatment ot Chronic Nasal Catarrh and Bronchitis......... 467
Treatment of Fracture of the Skull with Depression......... . 40
The Smoke Test for Tenements 45
Treatment of Vomiting in Pregnancy.. ........................... 46
Tanner Put to Open Shame by a Fasting Prisoner............... 81
The Hygiene of Trades.. ......... 96
The Continuous Inhalation of the Vapor of Slaking Lime in the Treatment of Membranous Laryngitis.................. 99
Treatment of Irites................. 103
The Moor Bath....................... 108
Thomasville as a Winter Resort 23
The Sea-side Hotel of the Future.......... ........... ........... 112
The Value of Graduated Pressure in the Treatment of the Vagina, Uterus, Ovaries and Other Appendages.........129, 195
Treatment of Epilepsy............ 170
The Toronto College and the Homœopaths..................... 178
The Surgeon-General's Annual Report................ .......... 180
The Treatment of Spasmodic Dysmenorrhœa..................... 730
Tricycles for Practitioners........ 746
Tuberculos s in Infants............ 748
Transfusion of Pure Water. .... 750
Things Worth Knowing........... 764
The Outlook of Medicine and Surgery.......... ........ ......... 718
Unalloyed P agiarism.............. 51
Ulcer of the Rectum............... 435
Urticaria .............................. 350
Vaccination of the Eye-lid ...... 155
Vomiting of Pregnancy........... 616
Valedictory............... ........... 436
Venereal and Common Warts... 242
Writer's Cramp...................... 174
Who Should be Medical Students ?........ ........................ 90
What is Homœopathy ?.......... 504
Woman and Her Ailments,...... 740
Yankee Brass........................ 761

